# METTL14 in tumor immunity: epitranscriptomic regulation and therapeutic potential

**DOI:** 10.3389/fimmu.2025.1709742

**Published:** 2025-10-14

**Authors:** Chunhong Li, Xiulin Jiang, Yixiao Yuan, Qiang Wang

**Affiliations:** ^1^ Department of Oncology, Suining Central Hospital, Suining, Sichuan, China; ^2^ Department of Systems Biology, City of Hope Comprehensive Cancer Center Biomedical Research Center, Monrovia, CA, United States; ^3^ Department of Gastrointestinal Surgical Unit, Suining Central Hospital, Suining, Sichuan, China

**Keywords:** METTL14, m^6^A methylase, tumor microenvironment, immune checkpoint, cancer immunotherapy

## Abstract

N^6^-methyladenosine (m^6^A) is the most abundant internal RNA modification, orchestrated by writers, erasers, and readers. METTL14, a key component of the m^6^A methyltransferase complex, acts as a structural scaffold that ensures substrate recognition and modification precision. Beyond this canonical role, METTL14 regulates multiple biological processes, including chromatin remodeling, transcriptional activity, and senescence-associated signaling. Recent studies highlight its pivotal function in tumor immunity: METTL14 shapes T cell differentiation, CD8^+^ T cell activation, and the activity of macrophages and NK cells, thereby remodeling the tumor immune microenvironment. Moreover, METTL14 directly modulates immune checkpoint pathways by regulating PD-1 and PD-L1 expression, linking epitranscriptomic control with immune escape and therapeutic resistance. Aberrant METTL14 expression correlates with tumor progression and immune evasion, underscoring its potential as a predictive biomarker and therapeutic target. Targeting METTL14, alone or in combination with immune checkpoint inhibitors, may provide novel strategies to enhance immunotherapy efficacy.

## 1 Introduction

N^6^-methyladenosine (m^6^A) is one of the most prevalent internal modifications in eukaryotic mRNA and long non-coding RNA, dynamically regulated by “writers”, “erasers” and “readers” (1).” As a reversible post-transcriptional modification, m^6^A governs multiple aspects of RNA metabolism, including splicing, nuclear export, stability, and translational efficiency, thereby exerting profound effects on cell fate determination and environmental adaptation (2–4). In the immune system, m^6^A modification has been shown to regulate both innate and adaptive immune responses (5, 6). For instance, it modulates dendritic cell (DC) antigen presentation and macrophage polarization by influencing interferon signaling and cytokine expression downstream of pattern recognition receptors. At the same time, m^6^A controls the expression of transcription factors and signaling molecules critical for CD4^+^ T cell lineage commitment, CD8^+^ T cell activation and exhaustion, and the maintenance of regulatory T cell (Treg) suppressive functions, ultimately shaping the strength and durability of immune responses (7). Collectively, these findings underscore m^6^A modification as a pivotal layer connecting genomic information with immune plasticity, playing a central role in sculpting the tumor immune microenvironment.

Within the m^6^A writer complex, METTL14 serves as an indispensable core component. The complex primarily consists of a METTL3–METTL14 heterodimer, with auxiliary cofactors such as WTAP ensuring its nuclear localization (8). Although METTL14 itself possesses minimal catalytic activity, it provides critical RNA substrate recognition and structural stabilization, thereby dictating the site selectivity and substrate specificity of m^6^A deposition (9). In immune cells, the functions of METTL14 exhibit strong cell type– and context-dependent features. In Tregs, METTL14-mediated m^6^A modification is essential for sustaining immunosuppressive function and homeostasis (10). Conversely, in CD8^+^ T cells, METTL14 regulates the expression of genes associated with effector function, cytokine production, and exhaustion, thereby influencing antigen-specific immune responses and therapeutic efficacy. These findings position METTL14 not only as a structural scaffold within the m^6^A machinery but also as a critical regulatory node governing immune cell fate and functionality.

In recent years, the advent of immune checkpoint inhibitors (ICIs) has revolutionized cancer therapy and markedly improved clinical outcomes across multiple malignancies (11). However, therapeutic responses remain highly heterogeneous, and only a subset of patients achieve durable benefit. The complexity and heterogeneity of the tumor immune microenvironment are recognized as major contributors to this variability (11). Against this backdrop, METTL14 and its mediated m^6^A modification have emerged as critical factors linking epigenetic regulation with tumor immunity. On one hand, METTL14 expression is closely associated with immune cell infiltration, immune-related gene expression, and patient prognosis, highlighting its potential as a predictive and prognostic biomarker (12). On the other hand, targeting METTL14 or modulating its downstream pathways may enhance the efficacy of ICIs and provide novel strategies to overcome therapeutic resistance (13). Notably, existing literature and reviews have largely focused on the role of METTL14 in tumorigenesis and cancer progression, while its contribution to tumor immune regulation remains relatively underexplored. Therefore, this review aims to systematically summarize the molecular mechanisms and biological functions of METTL14 in tumor immunity, and to further discuss its potential value and translational prospects in immunotherapy.

## 2 Biological functions and regulatory mechanisms of METTL14

To provide a foundation for understanding METTL14 impact on tumor immunity, this section will summarize its diverse biological functions and the multilayered regulatory mechanisms that govern its expression and activity, highlighting how these features position METTL14 as a central hub in cellular homeostasis and disease progression. METTL14 functions not only as a structural scaffold that maintains the stability and specificity of the m^6^A writer complex but also exerts a spectrum of m^6^A-independent roles (14). By regulating the senescence-associated secretory phenotype (SASP), chromatin states, and transcriptional activity, METTL14 critically influences cell fate decisions and homeostasis (15). Moreover, its expression and activity are subject to multilayered regulation, including epigenetic modifications, transcription factors, non-coding RNAs, and post-translational modifications, endowing METTL14 with remarkable dynamic plasticity across diverse physiological and pathological contexts. These molecular and network-level regulatory mechanisms not only underscore the multidimensional functionality of METTL14 but also establish its importance in tumorigenesis, immune modulation, and therapeutic resistance. Consequently, an in-depth exploration of its pathological roles and potential value in immunotherapy holds significant theoretical and translational relevance. In summary, METTL14 versatile functions and finely tuned regulatory mechanisms provide a mechanistic basis for its central role in immune regulation and tumor biology, setting the stage for a detailed examination of its specific molecular functions.

### 2.1 Role of METTL14 within the m^6^A writer complex

**Figure 1:**
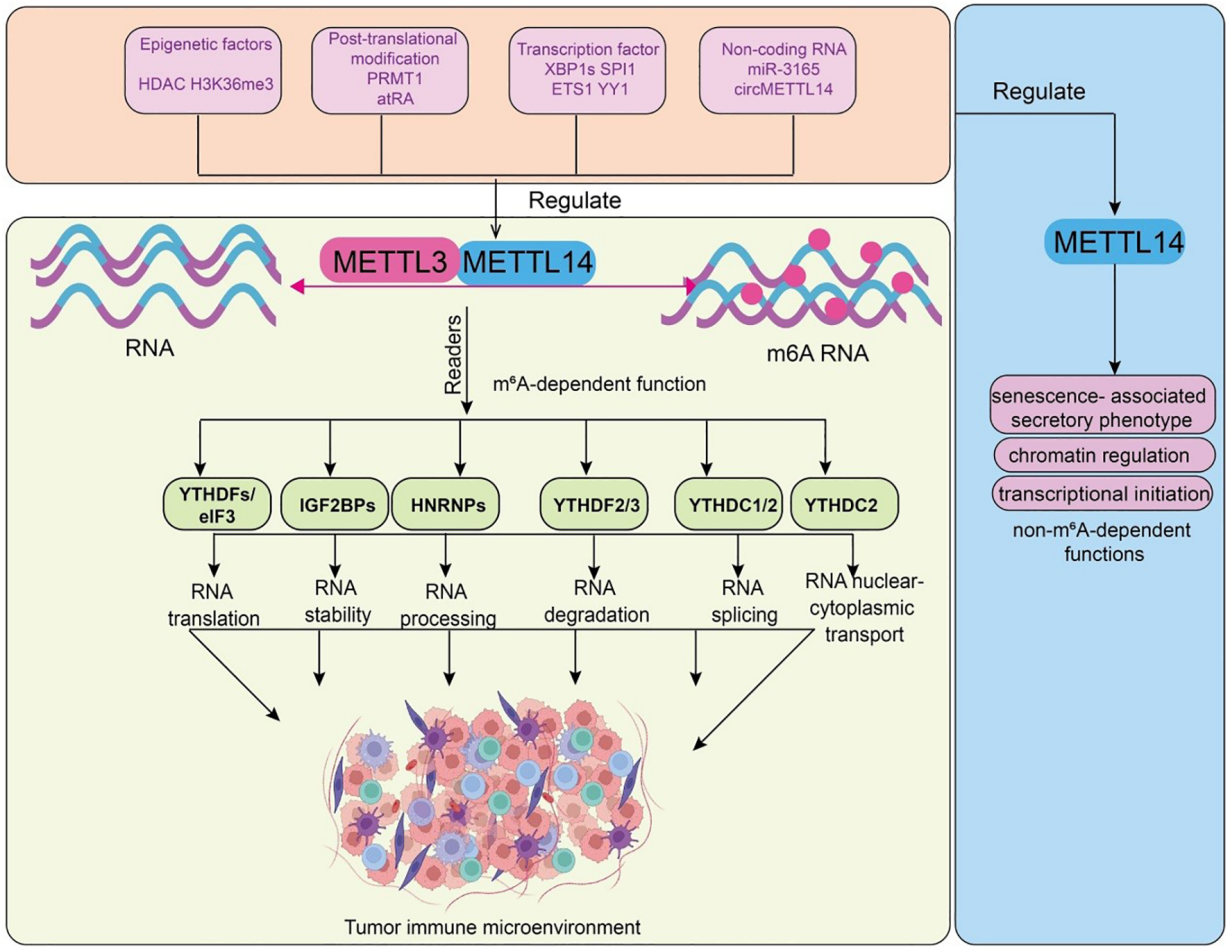
Functions and upstream regulatory mechanisms of METTL14. Epigenetic factors (HDAC, H3K36me3), post-translational modifications (PRMT1, atRA), transcription factors (XBP1s, SPI1, ETS1, YY1), and non-coding RNAs (miR-3165, circMETTL14) regulate the METTL3–METTL14 methyltransferase complex, leading to m^6^A RNA modification. m^6^A-modified RNAs are recognized by reader proteins, including YTHDFs/eIF3, IGF2BPs, HNRNPs, YTHDF2/3, YTHDC1/2, and YTHDC2, which regulate RNA translation, stability, processing, degradation, splicing, and nuclear–cytoplasmic transport. These events collectively reshape the tumor immune microenvironment. METTL14 non-m^6^A-dependent functions, including regulation of SASP, chromatin architecture, and transcriptional initiation, which contribute to its multifaceted influence on cell fate and disease.

This section focuses on METTL14 role as a structural scaffold and substrate recognition factor within the m^6^A methyltransferase complex, emphasizing how these features underpin its influence on RNA metabolism and downstream immune modulation. N^6^-methyladenosine (m^6^A) is the most abundant internal modification in eukaryotic mRNAs and various non-coding RNAs, dynamically regulated by “writers”, “erasers” and “readers”. Among them, the m^6^A methyltransferase complex (MTC) constitutes the central catalytic unit, composed of both catalytic and auxiliary subunits (1). As an essential component, METTL14 acts in concert with METTL3, WTAP, and other cofactors. Structurally, METTL14 forms a stable heterodimer with METTL3. While METTL3 harbors canonical catalytic activity that transfers a methyl group from S-adenosylmethionine (SAM) to adenosine, METTL14 lacks independent enzymatic activity due to evolutionary alterations in key catalytic residues within its methyltransferase domain (8) (**Figure 1**). Instead, METTL14 provides an extended RNA-binding interface and stabilizes METTL3 conformation, thereby enhancing substrate recognition efficiency and modification specificity. Functionally, METTL14 ensures the precision of m^6^A deposition. Its ability to recognize consensus motifs (RRACH) facilitates the enrichment of m^6^A at intron–exon junctions, the 3′ untranslated region (3′UTR), and regions near stop codons. This distribution pattern directly influences downstream RNA splicing, nuclear export, stability, and translation (16). Furthermore, through cooperation with cofactors such as WTAP, VIRMA, and RBM15/15B, METTL14 participates in guiding the localization of m^6^A marks to specific RNA regions, reinforcing the spatial specificity of the modification (16). In summary, METTL14 functions not as a catalytic core but as a structural scaffold and substrate recognition factor, stabilizing the MTC and coordinating auxiliary subunits to ensure high efficiency and specificity of m^6^A deposition. These structural and functional attributes provide the molecular basis for its pivotal role in immune regulation and disease progression. Overall, METTL14 structural and functional contributions to the m^6^A writer complex ensure precise RNA modification, providing the molecular foundation for its regulatory impact on gene expression, immune function, and disease progression.

### 2.2 m^6^A-independent functions of METTL14

Beyond its canonical role in m^6^A deposition, this section highlights METTL14 non-m^6^A-dependent functions, including regulation of SASP, chromatin architecture, and transcriptional initiation, which contribute to its multifaceted influence on cell fate and disease (**Figure 1**). Although METTL14 is best known as a key component of the m^6^A writer complex, recent studies have uncovered a range of biological functions independent of m^6^A modification, highlighting its multifunctionality. First, METTL14 regulates the senescence-associated secretory phenotype (SASP) in an m^6^A-independent manner, under conditions of without detectable changes in total m^6^A abundance (15). It upregulates the expression of SASP-related genes such as IL-6 and CXCL8, thereby promoting the secretion of cytokines by senescent cells (15). These secreted factors act in a paracrine manner to induce reprogramming or senescence in neighboring cells. For example, during the reprogramming of somatic cells into induced pluripotent stem cells (iPSCs), METTL14-driven SASP factor secretion by unsuccessfully reprogrammed cells enhances the reprogramming efficiency of adjacent cells (17). Second, METTL14 participates in chromatin regulation and transcriptional control, these chromatin regulatory effects were observed in cells expressing catalytically inactive METTL3, where global m^6^A levels remained unchanged. It binds to heterochromatic regions and recruits the histone demethylase KDM6B by recognizing the H3K27me3 mark, thereby reducing H3K27me3 levels and altering transcriptional activity. This function is critical for maintaining pluripotency and regulating differentiation in mouse embryonic stem cells (ESCs), independent of its role in m^6^A modification (17). Together, these findings reveal that METTL14 possesses non-m^6^A-dependent roles in regulating SASP gene expression, remodeling chromatin architecture, and facilitating transcriptional initiation. These discoveries not only broaden our understanding of METTL14 biological versatility but also highlight its roles in cell fate determination, senescence, and disease development. Together, these findings illustrate METTL14 m^6^A-independent versatility, reinforcing its significance in cell senescence, differentiation, and pathological processes relevant to tumor immunity.

### 2.3 Upstream regulatory mechanisms of METTL14 expression

This section will examine the hierarchical and interconnected upstream mechanisms that regulate METTL14 expression and activity, demonstrating how epigenetic, transcriptional, post-transcriptional, and post-translational layers collectively fine-tune METTL14 function in physiological and pathological contexts (**Figure 1**). The expression and activity of METTL14 are finely tuned not only by its role within the m^6^A methyltransferase complex but also by multilayered upstream mechanisms, which ensure precise regulation across physiological and pathological contexts. These mechanisms encompass chromatin modifications, transcription factor regulation, non-coding RNA mediation, and post-translational modifications, collectively determining METTL14 transcription, translation, and protein stability. At the chromatin level, epigenetic modifications directly influence the accessibility and transcriptional activity of the *METTL14* gene. In ocular melanoma, histone deacetylase inhibitors (HDACi) markedly increase global m^6^A levels by restoring histone acetylation at the *METTL14* promoter, reactivating its transcription (18). Upregulated *METTL14* subsequently enhances FAT4 expression through an m^6^A-YTHDF1-dependent pathway, exerting tumor-suppressive effects (18). In pulmonary arterial hypertension (PAH), SETD2-mediated H3K36me3 modification upregulates *METTL14* expression, leading to enhanced m^6^A deposition. Overexpressed METTL14 promotes pulmonary artery smooth muscle cell (PASMC) proliferation and exacerbates disease phenotypes in hypoxia-induced mouse models (19). Transcription factors also exert critical control. In breast cancer cells, endoplasmic reticulum (ER) stress induces XBP1s-dependent transcriptional activation of *METTL3/METTL14*, thereby elevating cellular m^6^A levels. In hematopoiesis, *METTL14* is highly expressed in hematopoietic stem/progenitor cells (HSPCs) and certain acute myeloid leukemia (AML) subtypes (t(11q23), t(15;17), t(8;21)), but its expression declines during myeloid differentiation. Importantly, SPI1 negatively regulates *METTL14*, forming a SPI1–METTL14–MYB/MYC axis essential for normal hematopoiesis and leukemogenesis (20). In neuroblastoma, *METTL14* expression is significantly elevated in high-risk patients and correlates with poor prognosis, with ETS1 and YY1 identified as upstream regulators (21). Non-coding RNAs further refine *METTL14* regulation. MicroRNAs, lncRNAs, and circRNAs modulate its expression either by directly targeting METTL14 mRNA or functioning as molecular sponges. For instance, *miR-3165* suppresses METTL14 expression in bladder cancer, promoting tumor progression via the *miR-3165–METTL14*–USP38 axis (22). In vascular endothelial inflammation underlying atherosclerosis, circMETTL14(11)S is highly expressed upon TNF-α stimulation and positively regulates *METTL14*, exacerbating inflammatory responses in human umbilical vein endothelial cells (HUVECs) (23). Post-translational modifications (PTMs) also play a pivotal role. Phosphorylation, ubiquitination, and acetylation directly affect METTL14 stability, subcellular localization, and interactions within the MTC. For example, PRMT1-mediated arginine methylation (R255me) enhances METTL14 binding to WTAP and RNA substrates, stabilizing MTC function, maintaining global m^6^A levels, and promoting endodermal differentiation in embryonic stem cells (24). During ER stress, accumulated unfolded/misfolded proteins induce METTL14 expression. METTL14 then promotes CHOP mRNA degradation via m^6^A modification at its 3′UTR, thereby suppressing pro-apoptotic gene expression and facilitating cell adaptation to stress (25). Mechanistically, the unfolded protein response (UPR) competes with the HRD1-ERAD pathway to prevent METTL14 ubiquitination and degradation, stabilizing its protein levels (25). In palatogenesis, environmental teratogen all-trans retinoic acid (atRA) induces aberrant upregulation of METTL14, elevating m^6^A levels in palatal mesenchymal cells (26). This disrupts proliferation and cell cycle gene expression, promoting cleft palate formation, which can be partially alleviated by siRNA-mediated METTL14 knockdown or inhibition of the m^6^A methyltransferase complex with SAH. Collectively, the upstream regulation of METTL14 is hierarchical and interconnected. These multilayered regulatory mechanisms not only maintain METTL14 homeostasis under normal conditions but also enable its dynamic responses to inflammation, immune signaling, and tumor microenvironmental changes. Understanding these regulatory pathways will provide crucial insights into the central role of METTL14 in tumor immune modulation and lay the foundation for developing METTL14-targeted therapeutic strategies. Collectively, these multilayered regulatory mechanisms ensure METTL14 homeostasis and dynamic responsiveness to cellular stress, inflammation, and tumor microenvironmental cues, highlighting their critical importance for METTL14-mediated immune modulation and providing a rationale for therapeutic targeting.

## 3 The role of METTL14 in the TME

With the rapid advancement of cancer immunotherapy, the TME has been increasingly recognized as a central determinant of therapeutic efficacy and resistance (27). As a core component of the m^6^A methyltransferase complex, METTL14 not only promotes tumor initiation and progression through post-transcriptional regulation of cancer cells, but also profoundly influences the differentiation, functional maintenance, and intercellular communication of immune cells. Accumulating evidence indicates that METTL14 exerts multi-level immune regulatory effects in the TME by modulating T cells, regulatory T cells (Tregs), tumor-associated macrophages (TAMs), and natural killer (NK) cells, thereby reshaping the immune landscape and regulating antitumor immunity. Together, these observations underscore METTL14 as a central hub linking epitranscriptomic regulation to tumor immune modulation, which is the core argument of this review. These findings not only deepen our understanding of tumor immune evasion mechanisms but also suggest that METTL14 may represent a promising target for improving immunotherapy sensitivity and overcoming immune resistance. As summarized in **Table 1**, METTL14 exhibits distinct targets, regulatory mechanisms, and functional effects across different tumors and immune cells.

**Table 1:** Key targets, regulatory mechanisms, and functional effects of METTL14 in different tumors or immune cells.

Effects	Cancer type/Immune cell	Upstream regulator	Direct target(s)/Pathway	m^6^A reader	Functional effect (Promote ↑/Suppress ↓)	Ref
Tumor promoting	CRC (T cells/TAMs)	–	*Ebi3* mRNA stabilization (↓m^6^A)	–	↑ *EBI3* → CD8^+^ T-cell dysfunction → immune evasion ↑	(30)
Tumor suppressing	CRC	–	*Stat1/Irf1* degradation	YTHDF2	↓ IFN-γ–Stat1–Irf1 signaling → CD8^+^ T-cell activity ↓ → PD-1 blockade resistance ↑	(12)
Tumor promoting	NSCLC	–	*circZNF548* suppression → exosomal *miR-7108-3p*	–	↓ CD8^+^ T-cell cytotoxicity ↑ tumor progression	(52)
Tumor suppressing	NSCLC	–	*PDCD1* mRNA degradation	–	↓ PD-1 expression → CD8^+^ T-cell activation ↑ (tumor growth ↓)	(53)
Tumor promoting	Breast cancer	–	–	–	METTL14 low → CD4^+^/CD8^+^ infiltration ↓ → poor prognosis ↑	(54)
Tumor promoting	Tregs	–	*FoxP3* expression; mTOR pathway inhibition	–	Maintains iTreg differentiation & suppressive function ↑	(32)
Tumor promoting	Tregs	–	*Sema4D* degradation	YTHDF2	Maintains Treg suppressive activity ↑; METTL14 loss ↑ Sema4D → ↑immune activation	(10)
Tumor promoting	ccRCC	–	*CCL5*	–	METTL14 low → Treg abundance ↑ (immune suppression ↑)	(55)
Tumor promoting	CRC	–	*Ebi3* stabilization (↓m^6^A)	–	↑ EBI3 → CD8^+^ T-cell dysfunction ↑	(30)
Tumor promoting	CESC	–	Glycolysis–lactate–PD-1 axis	–	Lactate ↑ PD-1 in TAMs → phagocytosis ↓, immune suppression ↑	(56)
Tumor promoting	ESCC	ZC3H13	*CXCL8* stabilization	–	TAM M2 polarization ↑, infiltration ↑	(36)
Tumor promoting	HCC	M1 exosomal *miR-628-5p* (↓METTL14)	*circFUT8/miR-552-3p/CHMP4B*	–	Drives HCC progression ↑	(57)
Tumor suppressing	NK cells	–	*Prf1, Gzmb* (effector genes)	–	Maintains NK maturation & cytotoxicity ↑	(58)
Tumor suppressing	iNKT cells	–	*Cish* suppression (TCR signaling)	–	Promotes iNKT development & cytokine production ↑	(39)
B-cell development promoting	B-cell development	–	IL-7 signaling; key TFs for pre-B transition	YTHDF2 (for IL-7 proliferation)	Promotes pro-B proliferation & large-to-small pre-B transition ↑	(40)

–not mentioned; ↑indicates promotion/activation; ↓indicates suppression/inhibition.

### 3.1 Regulation of T-cell infiltration and function

**Figure 2:**
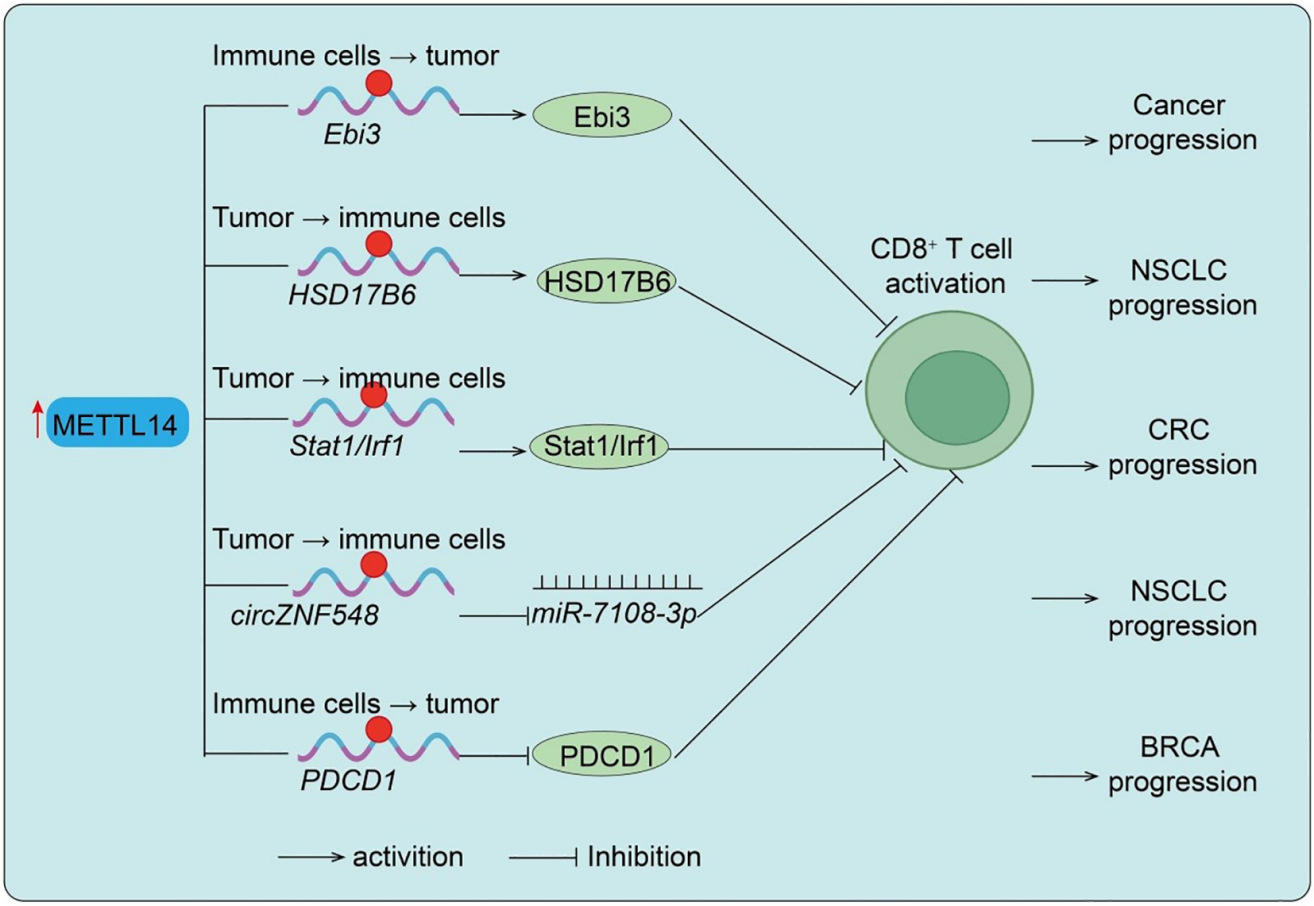
METTL14-mediated regulation of CD8^+^ T cell function in different cancers. Upregulation of METTL14 enhances m^6^A modification of target transcripts such as *Ebi3, HSD17B6, Stat1/Irf1*, and *circZNF548*, thereby modulating CD8^+^ T cell dysfunction, infiltration, activation, or killing capacity, ultimately influencing cancer progression in NSCLC and CRC. Conversely, reduced METTL14 expression leads to upregulation of *PDCD1*, suppressing CD8^+^ T cell activation and promoting BRCA progression. Tumor → immune cells indicate Tumor cell–initiated regulation of immune cells; Immune cells → tumor indicate Immune cell–intrinsic regulation of tumor cells.

T cells are the central executors of antitumor immunity, and their infiltration and cytotoxic activity are tightly regulated within the TME (28). This section illustrates how METTL14 directly modulates T-cell infiltration and cytotoxic function, highlighting its pivotal role as a mediator between m^6^A epitranscriptomic modification and adaptive antitumor immunity. Studies using CD4-Cre conditional knockout mice demonstrated that T cell–specific loss of *Mettl14* leads to spontaneous colitis characterized by increased inflammatory infiltration, elevated colon weight/length ratio, and enhanced Th1/Th17 cytokine expression (29). Mechanistically, *Mettl14* deficiency causes dysfunction of regulatory T cells (Tregs), marked by reduced RORγt expression and impaired iTreg differentiation, ultimately failing to suppress inflammatory responses (29). Rescue experiments confirmed that adoptive transfer of wild-type Tregs ameliorates colitis, while antibiotic treatment mitigates disease progression, highlighting the role of gut microbiota. In the tumor context, *METTL14* plays a crucial role in TAMs. In T-cell-specific METTL14 knockout mice, alterations in CD8^+^ T-cell cytotoxicity were observed, indicating that METTL14 acts primarily within T cells to modulate antitumor immunity. Macrophage-specific deletion of *METTL14* reduces m^6^A modification, thereby stabilizing *Ebi3* mRNA and increasing EBI3 protein expression. Elevated EBI3 drives CD8^+^ T cells toward dysfunction, diminishing their cytotoxicity and fostering tumor immune evasion. Blocking EBI3 restores CD8^+^ T-cell activity and enhances antitumor immunity (30) (**Figure 2**). Consistently, clinical colorectal cancer samples show a negative correlation between METTL14 expression/m^6^A levels and T-cell dysfunction. In lung cancer, METTL14 stabilizes *HSD17B6* mRNA through m^6^A modification, suppressing CD8^+^ T-cell infiltration and activation, ultimately facilitating tumor progression and impairing PD-1 blockade efficacy (**Figure 2**) (13). Similarly, in pMMR-MSI-L colorectal cancer, METTL14 promotes YTHDF2-dependent degradation of *Stat1/Irf1* mRNA, dampening IFN-γ–Stat1–Irf1 signaling and limiting CD8^+^ T-cell activity, which restricts PD-1 immunotherapy response (**Figure 2**). Additional evidence shows that *circZNF548*, downregulated in NSCLC and associated with favorable prognosis, enhances CD8^+^ T-cell cytotoxicity via exosomal *miR-7108-3p*, while METTL14 reduces *circZNF548* levels through m^6^A modification, thereby promoting tumor progression (**Figure 2**) (12). Interestingly, METTL14-mediated m^6^A-dependent degradation of *PDCD1* mRNA reduces PD-1 expression, maintaining CD8^+^ T-cell activation and restraining tumor growth (12) (**Figure 2**). Conversely, METTL14 loss elevates PD-1 levels, impairs T-cell function, and induces immunotherapy resistance. In breast cancer, METTL14 is frequently downregulated, correlating with ER-/PR-/triple-negative subtypes, poor prognosis, and advanced progression. Importantly, low METTL14 levels are positively associated with reduced infiltration of CD4^+^ and CD8^+^ T cells as well as neutrophils, underscoring its pivotal role in modulating TME and antitumor immunity. In summary, these findings demonstrate that METTL14 regulation of T-cell activity is a key mechanism by which epitranscriptomic modifications influence antitumor immunity, supporting its role as a critical node in the TME.

### 3.2 Regulatory T cells (Tregs)

**Figure 3:**
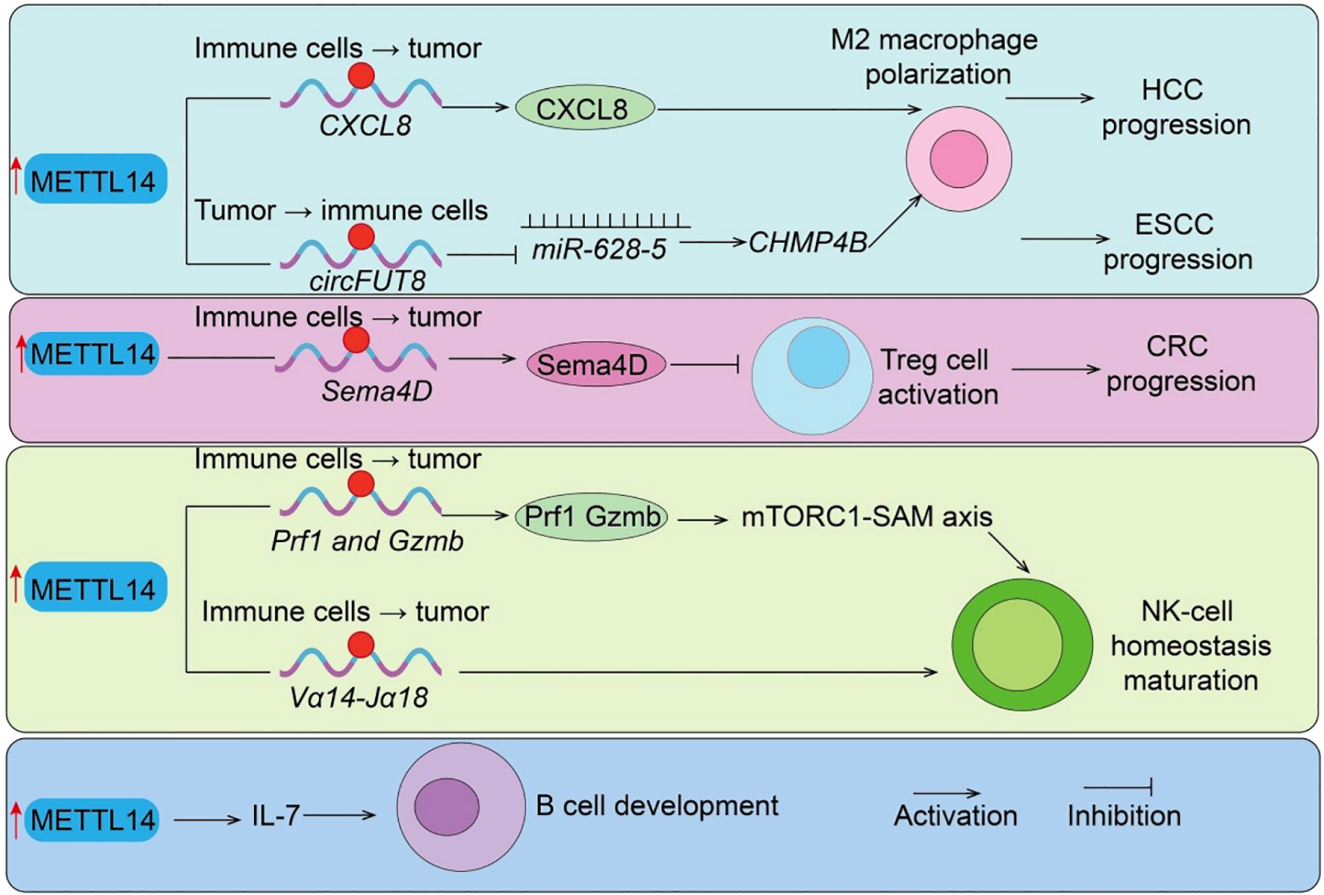
METTL14-mediated regulation of immunosuppressive cells and B cell development. METTL14 promotes tumor progression by regulating immune suppressive mechanisms: (i) *Sema4D* and *circFUT8/miR-628–5* axis drive Treg cell activation and M2 macrophage polarization in CRC and HCC; (ii) CXCL8 induces M2 macrophage polarization in ESCC; (iii)*Prf1/Gzmb*/Mtorc1-SAM axis regulate NK cells function. (iiii) METTL14/IL7 axis regulate B cells development. Tumor → immune cells indicate Tumor cell–initiated regulation of immune cells; Immune cells → tumor indicate Immune cell–intrinsic regulation of tumor cells.

Tregs are indispensable for immune tolerance and play a dual role in suppressing antitumor immunity within the TME (31). Here, we show that METTL14 is essential for Treg differentiation and suppressive function, connecting its epitranscriptomic activity to the modulation of immune tolerance and tumor immune escape. *In vitro* studies revealed that METTL14 expression is markedly upregulated in induced Tregs (iTregs). Silencing *METTL14* with siRNA reduced *FoxP3* expression, impaired differentiation, and elevated pro-inflammatory cytokines such as IFN-γ and IL-17a. Functional assays confirmed that *Mettl14* loss compromises iTreg suppressive capacity both *in vivo* (colitis mouse models) and *in vitro* (CFSE inhibition assays). Mechanistically, *Mettl14* deficiency activates the mTOR pathway (elevated p-mTOR and p-p70S6K), disrupting iTreg stability and immunosuppressive function (32). Further studies demonstrated that *Mettl14*-mediated m^6^A modification is essential for Treg expansion and immunosuppressive cytokine production (IL-10, TGF-β). Treg-specific knockout of *Mettl14* disrupts their suppressive capacity, leading to graft rejection, largely via SOCS pathway regulation (33). Moreover, *Mettl14*-YTHDF2–dependent degradation of *Sema4D* mRNA maintains Treg function, whereas METTL14 loss upregulates *Sema4D* (**Figure 3**), impairing immunosuppressive activity. Pharmacological inhibition of *Sema4D* restores Treg functionality and prolongs graft survival (10). Clinically, *Sema4D* expression negatively correlates with renal graft survival, supporting its role as a therapeutic target. In clear cell renal cell carcinoma (ccRCC), *METTL14* expression is inversely correlated with Treg abundance and positively associated with *CCL5* levels, suggesting a *METTL14/CCL5*/Tregs axis that shapes the tumor immune landscape (34). Collectively, these findings underscore METTL14 as a central regulator of Treg-mediated immune suppression, reinforcing its function as a molecular link between RNA methylation and immune modulation in the TME.

### 3.3 Recruitment and function of tumor-associated macrophages

Macrophages are highly plastic immune cells that critically influence tumor progression, and METTL14 has emerged as a regulator of TAM recruitment and function. This section highlights METTL14 role in orchestrating TAM behavior, providing evidence that its m^6^A-mediated regulation contributes to immune suppression and tumor progression, further connecting epitranscriptomic modifications to tumor immunity. TAM-specific loss of *METTL14* reduces global m^6^A levels, stabilizing *Ebi3* mRNA and increasing immunosuppressive *EBI3* expression. This drives CD8^+^ T-cell dysfunction and weakens antitumor responses, whereas *EBI3* blockade restores cytotoxic T-cell activity (30). Clinically, *METTL14* expression inversely correlates with CD8^+^ T-cell dysfunction in colorectal cancer. In cervical cancer, *METTL14* is overexpressed and enhances tumor glycolysis, producing lactate that upregulates PD-1 expression in TAMs (30). This suppresses phagocytosis and promotes an immunosuppressive TME. Functional and *in vivo* studies confirm the glycolysis–lactate–PD-1 axis as a critical mechanism by which METTL14 drives TAM-mediated tumor progression, identifying it as a potential therapeutic target. In hepatocellular carcinoma (HCC), M1 macrophage–derived exosomes deliver *miR-628-5p* to suppress METTL14, reducing *circFUT8* methylation and nuclear export (35). METTL14 otherwise promotes *circFUT8/miR-552-3p/CHMP4B* signaling, driving HCC progression (**Figure 3**), highlighting an interplay between macrophage exosomal miRNA and METTL14-mediated circRNA regulation (35). In esophageal squamous cell carcinoma (ESCC), ZC3H13 modulates METTL14/METTL3 nuclear transport and stabilizes *CXCL8* mRNA, driving M2 polarization and infiltration, thereby facilitating immune evasion (36). Taken together, these studies confirm that METTL14 modulates TAM recruitment, polarization, and immunosuppressive activity, illustrating another pathway by which epitranscriptomic regulation impacts antitumor immune responses.

### 3.4 NK cell function

Natural killer (NK) cells are innate lymphocytes essential for early antitumor responses (37). In this section, we demonstrate that METTL14-mediated m^6^A modification is crucial for NK and iNKT cell stability, maturation, and cytotoxic function, showing how epitranscriptomic regulation influences innate antitumor immunity. Short-term activation rapidly elevates m^6^A levels in NK cells, whereas this modification is suppressed within the TME (38). Single knockout of *METTL3 or METTL14* has minimal effect, but double knockout profoundly impairs NK-cell homeostasis, maturation, and cytotoxic function, underscoring their cooperative role. Mechanistically, m^6^A directly modifies effector genes such as *Prf1 and Gzmb*, regulating their expression, while the mTORC1-SAM axis drives rapid NK activation via m^6^A-dependent mechanisms (**Figure 3**). Similarly, m^6^A modification is indispensable for invariant NKT (iNKT) cell development (39). In T cell–specific *METTL14*-deficient mice, increased apoptosis of double-positive thymocytes reduces *Vα14-Jα18* rearrangement (**Figure 3**), resulting in decreased thymic and peripheral iNKT numbers (39). Residual iNKT cells exhibit increased apoptosis, impaired maturation, and weakened responses to *IL-2/IL-15* and TCR stimulation. Knockdown of METTL14 in mature iNKT cells upregulates Cish, suppresses TCR signaling, and reduces cytokine production. Overall, METTL14 ensures effective innate immune surveillance through m^6^A-dependent mechanisms, reinforcing its central role as a molecular hub linking epitranscriptomic modification with tumor immunity.

### 3.5 B cell development

RNA N^6^-methyladenosine (m6A) methylation, catalyzed by the METTL14 methyltransferase complex, plays a critical regulatory role in numerous biological processes (1). Studies have shown that deletion of *Mettl14* significantly reduces mRNA m6A methylation levels in developing B cells and severely impairs B-cell development in mice (40). Loss of *Mettl14* weakens interleukin-7 (*IL-7*)-induced pro-B cell proliferation and blocks the transition from large pre-B cells to small pre-B cells, while also causing abnormal expression of B-cell development–related genes (**Figure 3**). IL-7–induced pro-B cell proliferation depends on the cytoplasmic m6A reader YTHDF2, which suppresses a subset of transcripts, whereas the block in large-to-small pre-B cell transition is independent of either YTHDF1 or YTHDF2 and instead results from the failure to properly upregulate key transcription factors (40). Overall, this study highlights the essential regulatory roles of RNA m6A methylation and its reader proteins in early B-cell development.

## 4 METTL14 and immunotherapy

To provide a clear framework for the following sections, this part will highlight how METTL14, as a core component of the m^6^A **“**writer**”** complex, regulates immune checkpoint molecules and the tumor immune microenvironment, thus serving as a central node linking epitranscriptomic regulation to antitumor immunity. In recent years, the clinical application of tumor immunotherapies, particularly immune checkpoint inhibitors (ICIs), has significantly improved the prognosis of certain cancer patients. However, their efficacy remains limited by immune evasion and resistance mechanisms. RNA N^6^-methyladenosine (m^6^A) modification, as a key layer of epitranscriptional regulation, has increasingly been recognized as a critical determinant of tumor immune microenvironment remodeling and immunotherapy response. As a core component of the m^6^A “writer” complex, METTL14 not only regulates the expression of immune checkpoint molecules such as *PD-1 and PD-L1* to modulate T cell function and immune escape, but also interacts with multiple non-canonical signaling pathways, thereby profoundly influencing tumor sensitivity to immunotherapy. In summary, the mechanistic and therapeutic significance of METTL14 positions it as a pivotal link between epitranscriptomic regulation and tumor immune responses, setting the stage for a deeper discussion of its role in immunotherapy efficacy.

### 4.1 Regulation of immune checkpoint inhibitor efficacy

This section will focus on how METTL14 modulates *PD-1/PD-L1* and related molecules to regulate T cell function and tumor immune evasion, emphasizing its critical role in immunotherapy efficacy. Evidence has shown that METTL14 promotes m^6^A-dependent degradation of *PDCD1* mRNA, thereby downregulating PD-1 expression, sustaining CD8^+^ T cell activation, and suppressing tumor progression. Conversely, *METTL14* deficiency results in elevated PD-1 levels, impaired T cell function, and resistance to immunotherapy. This highlights the *METTL14*–PD-1 axis as a critical regulatory pathway and suggests that targeting *METTL14* in combination with PD-1 blockade may hold translational value. In glioblastoma (GBM), METTL14 is highly expressed and enhances *PD-L1* stability by promoting its m^6^A modification. Knockdown of *METTL14* significantly suppresses GBM proliferation, migration, and immune evasion while slowing tumor growth in murine models (41) (**Figure 3**). Mechanistically, METTL14-mediated m^6^A modification stabilizes PD-L1 mRNA in an IGF2BP2-dependent manner. Rescue experiments confirmed that PD-L1 overexpression reverses the inhibitory effect of METTL14 knockdown, underscoring PD-L1 as a key downstream effector (41). Thus, METTL14 drives GBM progression and immune escape by stabilizing PD-L1 via an IGF2BP2-dependent mechanism. In cholangiocarcinoma (CCA), an m^6^A–METTL14–*Siah2–PD-L1* axis has been identified. METTL14 promotes m^6^A deposition on the 3′UTR of *Siah2* mRNA, enhancing its YTHDF2-dependent degradation and ultimately upregulating *Siah2* expression (42). *Siah2* directly interacts with PD-L1, regulating its stability through K63-linked ubiquitination. Knockdown of *Siah2* maintains PD-L1 expression in tumor cells, markedly impairing T cell proliferation and cytotoxicity (42) (**Figure 3**). Clinical analysis confirmed the presence of this axis in CCA tissues and demonstrated that patients with low *Siah2* expression were more responsive to PD-1 blockade. Collectively, these findings reveal a novel mechanism whereby METTL14 regulates PD-L1 stability via *Siah2*, providing new therapeutic insight for CCA immunotherapy. In hepatocellular carcinoma (HCC), METTL14 plays a key role in immune escape. In orthotopic Hepa1–6 models, lipopolysaccharide (LPS) stimulation significantly upregulated PD-1 and PD-L1 expression. Mechanistic studies showed that LPS enhanced METTL14 expression, which in turn stabilized the *lncRNA MIR155HG* through m^6^A modification in an ELAVL1 (HuR)-dependent manner. *MIR155HG* acted as a competing endogenous RNA (ceRNA) regulating the *miR-223/STAT1* axis, thereby further increasing *PD-L1* expression. This *LPS–METTL14–MIR155HG–PD-L1* axis was validated in HepG2 xenografts and was particularly prominent in HCC with cirrhosis, suggesting a novel m^6^A-dependent lncRNA regulatory pathway contributing to HCC immune escape (43). In non-small cell lung cancer (NSCLC), *KCTD10* expression is significantly downregulated in tumor tissues. Functional assays revealed that *KCTD10* overexpression effectively suppressed tumor progression both *in vitro* and *in vivo*. Mechanistically, *KCTD10* interacted with β-catenin via its BTB domain, promoting β-catenin K48-linked ubiquitination and degradation, thereby suppressing downstream PD-L1 expression (44). Importantly, combined *KCTD10* overexpression and PD-1 blockade exhibited a pronounced synergistic effect in suppressing lung cancer progression and brain metastasis. Notably, METTL14 directly enhanced the stability of *KCTD10* mRNA via m^6^A modification within its coding sequence in a YTHDF2-dependent manner (44). Taken together, *KCTD10* suppresses lung cancer progression and immune escape via the β-catenin/PD-L1 axis, and its expression is tightly regulated by METTL14-dependent m^6^A modification, highlighting its potential as a therapeutic target. Taken together, these findings underscore METTL14 as a master regulator of immune checkpoint signaling and tumor immune escape, providing a mechanistic rationale for targeting METTL14 to enhance immunotherapy responses.

### 4.2 Potential of METTL14 inhibitors in combination immunotherapy

**Figure 4:**
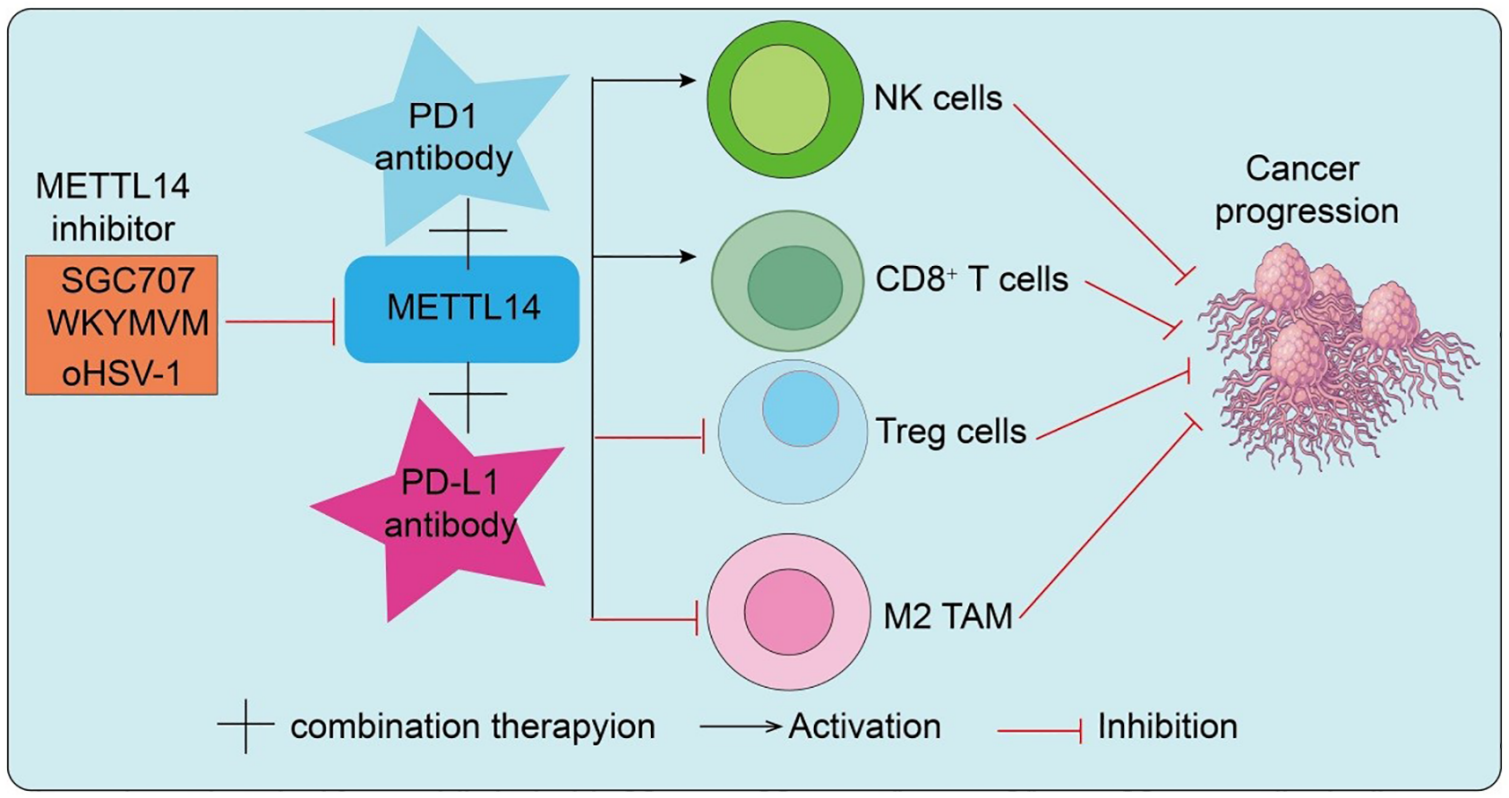
METTL14 inhibitors synergize with PD-1/PD-L1 antibodies to remodel the tumor immune microenvironment and suppress cancer progression.

This section will explore the therapeutic potential of targeting METTL14 with inhibitors, emphasizing how modulating METTL14 activity can synergize with immune checkpoint blockade and overcome resistance, further demonstrating METTL14 role as a key link between epitranscriptomic regulation and antitumor immunity. With the rapid development of RNA epigenetic therapeutics, the METTL14-centered m^6^A methyltransferase complex has emerged as a novel druggable target. Preclinical studies have demonstrated that pharmacological inhibition of METTL14 reduces global m^6^A levels, destabilizes oncogenic transcripts, and suppresses malignant tumor progression. More importantly, because METTL14-mediated m^6^A modification enhances PD-L1 expression and promotes an immunosuppressive microenvironment, inhibition of METTL14 may not only directly impair tumor proliferation but also downregulate PD-L1 expression to improve T cell–mediated antitumor immunity. Therefore, combining METTL14 inhibitors with PD-1/PD-L1 ICIs offers synergistic therapeutic potential and may help overcome resistance to monotherapy in subsets of patients. Interestingly, viral infection studies provide additional mechanistic insights. During early HSV-1 infection, the immediate-early protein ICP0 interacts with METTL14 and targets it for ubiquitination at K156 and K162, leading to proteasomal degradation and reduced cellular m^6^A levels (45). Normally, METTL14 stabilizes *ISG15* mRNA via IGF2BP3, contributing to antiviral defense. By degrading METTL14, HSV-1 suppresses this pathway to facilitate immune evasion (45). Remarkably, METTL14 inhibition enhances the efficacy of oncolytic HSV-1 (oHSV-1) in glioma, suggesting that the METTL14–*ISG15* axis is both a viral immune checkpoint and a therapeutic target to potentiate oHSV-1 antitumor activity. In endometrial carcinoma, PRMT3 regulates METTL14 through arginine methylation. Pharmacological inhibition of PRMT3 (e.g., SGC707) relieves this repression, enhances METTL14 expression and m^6^A–YTHDF2–dependent modification, destabilizes GPX4 mRNA, and induces lipid peroxidation and ferroptosis (46). Functionally, PRMT3 inhibition sensitizes endometrial cancer cells to PD-1 blockade, cisplatin, and radiotherapy, highlighting PRMT3 as a novel therapeutic target that indirectly modulates METTL14 activity to enhance ferroptosis and immunotherapy efficacy. Moreover, combined inhibition of METTL3/METTL14 with paclitaxel (PTX) demonstrated potent synergistic antitumor effects in breast cancer cells and xenograft models. Mechanistic studies revealed that METTL14 stabilizes *E2F1* mRNA through an m^6^A–IGF2BP2–dependent mechanism, contributing to resistance against CDK4/6 inhibitors (CDK4/6i) (47). A novel small-molecule inhibitor, WKYMVM, effectively reversed CDK4/6i resistance and significantly enhanced therapeutic efficacy when delivered via liposomal formulations. As shown in **Figure 4**, the combination of METTL14 inhibitors with PD-1/PD-L1 antibodies synergistically remodels the tumor immune microenvironment, enhancing antitumor immunity and effectively suppressing cancer progression. This highlights the potential of targeting METTL14 as a strategy to improve immunotherapeutic efficacy (**Figure 4**). Overall, these studies highlight METTL14-centered therapeutic strategies as a promising avenue to overcome immunotherapy resistance and potentiate antitumor immunity, reinforcing METTL14 central position at the intersection of epitranscriptomic regulation and immune modulation.

## 5 Clinical significance and perspectives

With growing evidence of the immunoregulatory role of m^6^A modification, the multifaceted functions of METTL14 within the tumor immune microenvironment (TME) are being progressively unraveled, underscoring its translational value. As a core component of the m^6^A writer complex, METTL14 expression and activity are tightly associated with immune cell function and may critically determine patient responsiveness to immunotherapy, thereby holding great promise in precision oncology (48, 49). First, METTL14 may serve as a predictive biomarker for immunotherapy. Multiple studies demonstrate that m^6^A modification levels are closely linked to the efficacy of PD-1/PD-L1 blockade and other ICIs. METTL14 expression may influence immune cell infiltration, antigen presentation, and cytotoxic lymphocyte activity. Thus, assessing METTL14 status could provide a valuable indicator for predicting therapeutic response and prognosis. Second, targeting METTL14 or its downstream signaling pathways offers novel therapeutic opportunities. Direct modulation via small-molecule inhibitors, RNA interference, or genome editing—or indirect targeting of pathways such as T cell activation and dendritic cell function—could enhance immunotherapy sensitivity and improve clinical outcomes. Importantly, METTL14 exhibits “context-dependent” and “double-edged sword” characteristics: while it may potentiate antitumor immunity in some settings, it could promote immune suppression in others. This duality poses significant challenges for clinical translation and highlights the need for context-specific therapeutic strategies.

Future directions warrant particular attention. (i) The role of METTL14 in phase separation may regulate RNA–protein condensate assembly, influencing transcriptional and translational efficiency in immune cells. Recent studies suggest that liquid–liquid phase separation (LLPS) serves as a key mechanism for the spatial and temporal organization of biomolecules, including RNA, proteins, and chromatin-associated factors. In the context of immunity, LLPS can facilitate the formation of membrane-less condensates such as immunological synapses, transcriptional hubs, or stress granules, thereby concentrating signaling molecules and enhancing the efficiency of immune responses (50). For instance, phase-separated condensates may regulate T-cell receptor (TCR) signaling by clustering key kinases and adaptor proteins, promoting rapid phosphorylation cascades and downstream cytokine production. Similarly, LLPS can modulate the localization and activity of RNA-binding proteins or m^6^A readers/writers like METTL14, affecting mRNA stability and translation of immune-related genes (51). Collectively, these observations indicate that phase separation provides an additional layer of epitranscriptomic and signaling regulation, enabling precise control of innate and adaptive immune functions within the tumor microenvironment. (ii) Its involvement in chromatin modification and 3D genome architecture suggests functions beyond canonical RNA methylation, potentially linking METTL14 to super-enhancer–mediated regulation of immune gene activity. The three-dimensional organization of the genome is increasingly recognized as a critical determinant of gene expression and cellular identity, including in immune cells. Chromatin looping, topologically associating domains (TADs), and enhancer–promoter contacts can dynamically regulate immune gene accessibility and transcriptional programs. For example, spatial proximity between interferon-stimulated gene clusters and super-enhancers can potentiate rapid antiviral or antitumor responses. Similarly, the 3D genome may influence T-cell differentiation or regulatory T-cell function by modulating long-range interactions that control cytokine or transcription factor loci, such as FoxP3 or Stat1. Moreover, epitranscriptomic modifiers, including METTL14 (51), may interact with specific chromatin regions to coordinate m^6^A deposition with 3D chromatin architecture, thereby linking RNA modification to gene regulatory landscapes in immune cells. These insights highlight 3D genomics as a forward-looking mechanism for fine-tuning immune responses and shaping tumor-immune interactions. (iii) Preclinical and clinical studies combining METTL14 knockdown or inhibition with ICIs or other immunotherapies could yield synergistic effects, paving the way for innovative combination strategies. In conclusion, research on METTL14 in tumor immunity remains in rapid evolution. As both a biomarker and therapeutic target, METTL14 presents exciting opportunities alongside complex challenges. Future mechanistic studies and large-scale clinical validation will be essential to bridge the gap from bench to bedside and to realize the full potential of METTL14 in cancer immunotherapy.

## 6 Limitations and context-dependent roles of METTL14 in tumor immunity

Despite extensive evidence highlighting the pivotal role of METTL14 in regulating tumor progression and immune cell function, several limitations must be acknowledged to provide a balanced perspective. First, the functions of METTL14 are highly context-dependent, varying across tumor types, immune cell subsets, and microenvironmental conditions. For instance, METTL14 may promote CD8^+^ T-cell dysfunction in colorectal cancer through stabilization of Ebi3 mRNA, yet enhance T-cell activation in other contexts by facilitating PDCD1 mRNA degradation (30). Similarly, its regulatory effects on Tregs, TAMs, and NK cells are influenced by local cytokine milieu, metabolic conditions, or epigenetic landscapes. These differences may arise from heterogeneous expression of m^6^A readers (e.g., YTHDF2, IGF2BP2), co-factors, and signaling intermediates, as well as the interplay between m^6^A-dependent and -independent functions such as chromatin remodeling or transcriptional regulation. Second, experimental limitations exist in many studies. Most mechanistic insights are derived from murine models or *in vitro* systems, which may not fully recapitulate the human tumor microenvironment. Additionally, global manipulation of METTL14 (e.g., knockout or knockdown) may obscure cell-type-specific effects, making it challenging to delineate precise molecular mechanisms. Contextual variables such as tumor stage, mutational burden, and microbiome composition may further modulate METTL14 functions, yet remain underexplored in current research.

Third, these context-dependent roles pose significant challenges for clinical translation. The dual and sometimes opposing functions of METTL14 across tumors and immune cell types complicate its application as a universal biomarker or therapeutic target. Systemic targeting of METTL14 could inadvertently disrupt immune homeostasis or impair anti-tumor immunity in specific contexts. Therefore, patient stratification based on tumor type, immune cell composition, and METTL14 expression patterns, along with the development of cell-type-specific delivery systems, will be critical for safe and effective therapeutic interventions. Moreover, the integration of emerging concepts such as phase separation and 3D genome architecture may provide additional layers of regulatory insight, potentially guiding more precise manipulation of METTL14 in the tumor immune microenvironment. Collectively, while METTL14 represents a promising target in cancer immunotherapy, future studies should carefully consider its context-dependent functions, mechanistic complexity, and translational constraints to fully realize its therapeutic potential.
